# Proof of concept for multiplex detection of antibodies against *Chlamydia* species in chicken serum using a bead-based suspension array with peptides as antigens

**DOI:** 10.1186/s13567-023-01159-9

**Published:** 2023-04-04

**Authors:** Fimme J. van der Wal, René P. Achterberg, Jeanet A. van der Goot, Annemieke Dinkla, Ruth Bossers-de Vries, Conny van Solt-Smits, Alex Bossers, Marloes Heijne

**Affiliations:** Wageningen Bioveterinary Research, P.O. Box 65, 8200 AB Lelystad, Netherlands

**Keywords:** Chicken, serum antibodies, serology, *Chlamydia*, peptide, antigen, suspension array

## Abstract

The available differentiating tests for *Chlamydia* are based on detection of genetic material and only give information about the actual infection status, but reveal nothing of past infections. As the use of serological methods increases the window of detection, the goal of this study was to investigate if it is possible to develop a differentiating serological test for antibodies against *Chlamydia* species in chicken sera. Focus was on *C. psittaci*, *C. gallinacea*, and two closely related species, i.e. *C. abortus* and *C. avium*. To enable differentiating serology, a bead-based Luminex suspension array was constructed, using peptides as antigens, derived from known immunoreactive *Chlamydia* proteins. For the majority of these peptides, species-specific seroreactivity in mammalian sera has been reported in literature. The suspension array correctly identified antibodies against various *Chlamydia* species in sera from experimentally infected mice, and was also able to differentiate between antibodies against *C. psittaci* and *C. gallinacea* in sera from experimentally infected chickens. In field sera, signals were difficult to interpret as insufficient sera from experimentally infected chickens were available for evaluating the seroreactivity of all peptides. Nevertheless, results of the suspension array with field sera are supported by published data on the occurrence of *C. gallinacea* in Dutch layers, thereby demonstrating the proof of concept of multiplex serology for Chlamydial species in poultry.

**Supplementary Information:**

The online version contains supplementary material available at 10.1186/s13567-023-01159-9.

## Introduction

*Chlamydia* is an expanding genus of Gram-negative obligate intracellular bacteria, currently containing 14 different species [1], of which some were discovered only in the last decade [2], and with proposed new species pending [3]. Each species has a preferred host or host range, and many cannot only cause disease in their preferred hosts, but also have zoonotic potential [4]. The most infamous zoonotic species is *Chlamydia psittaci*, which has been detected in hundreds of bird species [5] including poultry [6]. In birds, *C. psittaci* infections can result in mild to severe disease, depending on the avian species and the *Chlamydia* strain, whereas an infection in humans can lead to severe pneumonia, i.e. psittacosis [7].

In the past, *C. psittaci* was considered the main *Chlamydia* species in poultry, including chickens [6–8]. More recent observations indicate that in chickens *Chlamydia gallinacea* is the most prevalent *Chlamydia* species [9–11]. Apart from a reduction in weight gain, infections with *C. gallinacea* are asymptomatic [9]. Conclusive evidence for zoonotic potential has not been presented yet, but cases of atypical pneumonia in slaughterhouse personnel coinciding with the occurrence of *C. gallinacea* have been reported [12].

Since *C. gallinacea* and *C. psittaci* can infect chickens and are likely to differ in epidemiology and zoonotic potential, it is important to have diagnostic tools available that allow detection of, and distinction between, these two species, both from a public health perspective as from an economic point of view. Current discriminatory tests rely completely on molecular methods [13] and only give information about the present infection status of animals. Discriminatory serology, however, will have added value as serology is a very easy way of monitoring (past) occurrence of any pathogen in animal production chains. At the time of writing, for *Chlamydia* serology in birds only a non-discriminatory ELISA is available [13, 14].

The goal of this study is to investigate if it is possible to develop a serological test that, in chicken serum, can differentiate between antibodies against different *Chlamydia* species, particularly *C. psittaci* and *C. gallinacea*, using peptides as antigens. Sequences of *Chlamydia*-derived peptides are available from the literature: a considerable number of sequences have been identified through comprehensive bio-informatics and subsequent screening with sera from infected mice [15, 16]. A subset of these peptides has successfully been used in arrays to differentiate between antibodies against various *Chlamydia* species in multiple mammalian hosts [17]. In the current study, we investigated whether peptides that have been used as antigens in mammalian serology also allow detection and differentiation of antibodies against *Chlamydia* species in chicken sera. To this end, a multiplex assay for antibody detection in chicken sera was developed, using *Chlamydia*-derived peptides as antigens in a Luminex suspension array, analogous to earlier work on detection of antibodies against (other) pathogens in sera from poultry and swine [18–20]. Here, protocols for peptide-based multiplex serology in chicken sera are given, including the use of serum-specific cutoffs for normalization, based on an internal control. Results obtained with sera from experimentally infected mice and chickens and with chicken sera from various types of farms, are discussed in detail. The proof of concept multiplex suspension array for antibodies against Chlamydial species in poultry correctly identifies *Chlamydia* antibodies in sera from experimentally infected animals, and observations in field samples are supported by published data on the occurrence of *C. gallinacea* in Dutch layers.

## Materials and methods

### Sera

Pooled sera from mice infected with different *Chlamydia* species [15, 16] were donated by Bernhard Kaltenboeck (Auburn University, Alabama, USA) and shipped by Christiane Schnee (Friedrich-Loeffler-Institut, Jena, Germany).

Sera from chickens experimentally infected with *C. gallinacea* or *C. psittaci* were available from various in-house experiments. From a published experimental infection with *C. gallinacea* NL_G47 [21, 22], sera were used from day 0, 14, 28, and 35 after oral inoculation of 5-week-old PCR-negative chickens (*n* = 8). From an unpublished experimental infection with *C. psittaci* 6BC, sera were available from day 0, 14, and 21 after exposure, and PCR data was available from day 4 (throat and cloaca swabs) and day 21 (airsacs, lungs and spleens) after exposure. This experiment was performed in essence as described elsewhere [23]. In short, 6-week-old SPF laying hens were placed for 1 h in an insulator in which 10 mL of a suspension with live *C. psittaci* 6BC (10^6^ TCID_50_ per mL) was sprayed with a nebulizer. After infection, chickens were held in an open pen on sawdust bedding.

From a collection of ca. 2400 field sera from 2015, originating from the Dutch program for avian influenza monitoring performed by Royal GD, 120 sera from six farms were selected for testing. The collection contained 20 sera per farm, of which two farms of each of three types of farms, i.e. with conventional layers, free range layers, or broilers, were taken. The farms were selected simply by order of appearance in the collection, so the first appearing two serum sets from each farm type were picked.

### Antigens

For ELISA, two commercially available antigens were used, i.e. *C. abortus* and *Chlamydia trachomatis* isolated from elementary and reticular bodies, inactivated by heat treatment and sonication (Virion/Serion, Würzburg, Germany).

For the suspension array, peptides were used as antigens. Peptide sequences derived from *Chlamydia* species that are relevant for poultry (*C. psittaci* and *C. gallinacea*) and from two genetically closely related species (resp. *Chlamydia abortus* and *Chlamydia avium*) were taken from literature. A selection was made based on signal strength and specificity as described [15–17]. In addition, for two published *C. gallinacea*-derived peptides, corresponding peptide sequences from two Dutch field isolates, NL_G47 and NL_F725 [24], were selected. All peptide sequences are listed in Table 1. Peptides ranging from 16 to 40 amino acids were synthesized on 50 µmol scale and pHPLC purified (Pepscan, Lelystad, The Netherlands). The peptides contained an N-terminal biotine separated from the peptide moiety by a spacer, consisting of five units of 8-amino-3,6-dioxaoctanoic acid, as used earlier [19, 20]. A compound that served as negative control (NC) was synthesized in exactly the same way, but without a peptide moiety (Table 1), and was used to determine assay background in each test for each serum.

**Table 1:** **Peptides used as antigens**

Species	Peptide name	Description^a^	Sequence	Length	Source
*C.gallinacea*	pCga001	Cga_08DC63_OmpA_326-345	NPSFLGSADAQATLVDSVQI	20	[15, 17]
	pCga002	Cga_08-1274/3_IncA_297-326	SEAATSTSTPEGETSETKEGEEDSSVVEFD	30	[15, 16]
	pCga003	Cga_08DC63_IncA_303-326	TSTPEGETSETKEGEEDSSVVEFD	24	[17]
	pCga008	C. gal G47_OmpA	NPTFSGGAVPQTGGTGSVVDVVQI	24	This study
	pCga009	C. gal F725_OmpA	NPSFLGEANAQAKLVDSVQI	20	This study
	pCga010	C. gal G47_IncA	SEAATSTSTPEGETSETKEGEEDSSVIEFD	30	This study
	pCga011	C. gal F725_IncA	SEAATSTSTPEGETSETKEGEEDSSVIDFD	30	This study
*C. psittaci*	pCps001	Cps_02DC15_OmpA_158-181	LVGLIGFSAASSISTDLPTQLPNV	24	[15–17]
	pCps002	Cps_02DC15_IncA_329-352	ADQGDLRDPSGDRYGGWGAQSSYR	24	[17]
	pCps009	Cps_02DC15_OmpA_250-265	ASSNFPLPITAGTTEA	16	[16]
	pCps010	Cps_02DC15_OmpA_329-352	SLIGSTTALPNNSGKDVLSDVLQI	24	[17]
	pCps013	Cps_02DC15_PmpD_1053-92	DPNAKPAEKIESPTSKVYYSAYDPVKNPGKKTLADINSIG	40	[16]
	pCps014	Cps_02DC15_IncA_321-360	SLTSTTETADQGDLRDPSGDRYGGWGAQSSYRLSPSVTMS	40	[15, 16]
	pCps015	Cps_02DC15_CT618_105-134	YEVDSATGSFKIVTKNIQKPNGEVEIVSSR	30	[15, 16]
	pCps016	Cps_02DC15_CT618_189-228	CGAVDDVISIVSTLRSTDFDPSYEDLVQRRVTLREKFFSL	40	[15, 16]
*C.abortus*	pCab001	Cab_S26/3_OmpA_89-104	PTGTAAANYKTPTDRP	16	[16]
	pCab002	Cab_S26/3_OmpA_153-176	NLVGLIGVKGSSIAADQLPNVGIT	24	[15–17]
	pCab003	Cab_S26/3_OmpA_158-173	GVKGSSIAADQLPNV	15	[17]
	pCab004	Cab_S26/3_PmpD_1064-1087	PTSNVYYSAHESVKQPENKTLADI	24	[17]
	pCab005	Cab_S26/3_IncA_324-353	STAVTEHADIPRDPNRDPRGGRGGQSSPSV	30	[15, 17]
*C. avium*	pCav001	Cav_10DC88_IncA_305-328	ESTPVEAPESKEEAKDTAEVAAEG	24	[17]
	pCav002	Cav_10DC88_IncA_299-328	TVEGAAESTPVEAPESKEEAKDTAEVAAEG	30	[15]
	pCav003	Cav_10DC88_IncA_325-348	AAEGSGSTEESKGKEDDKSGDKKE	24	[17]
	pCav004	Cav_10DC88_IncA_319-348	KDTAEVAAEGSGSTEESKGKEDDKSGDKKE	30	[15, 16]
n.a	NC	Biotin-spacer-(no peptide)-amide	n.a	0	this study

Peptide sequences were selected for four *Chlamydia* species based on literature and on sequences of Dutch isolates G47 and F725. Partial alignments can be found in Tables 2 and 3.

P: purified peptide; Cga, Cps, Cab, and Cav refer to the *Chlamydia* species (see first column); NC: negative control; n.a.: not applicable.

^a^*C. gallinacea* strain 08DC63 and 08-1274/3 are the same.

### ELISA

An in-house ELISA with a mix of *C. abortus* and *C. trachomatis* antigens (Virion/Serion) was used to detect antibodies against *Chlamydia* species. In previous work, this ELISA was used to detect an increase in antibodies in *C. gallinacea* infected chickens, but a cutoff has not been established [21]. Where relevant, results are given and expressed as optical density (OD) values.

### Bead sets with immobilized peptides

Biotinylated peptides and the compound that serves as negative control (NC) were immobilized as described before [19], using 5 × 10^5^ avidin coated paramagnetic beads (MagPlex-Avidin microspheres; Luminex, Den Bosch, The Netherlands). The biotinylated compounds were used at a final concentration of 4000 nM. After binding of these compounds to avidin-coated beads, the beads were washed and blocked with biotin [19].

In total, 10 spectrally distinct bead sets were used to assemble three different bead mixes. Each bead mix contained a bead set with the NC and up to 9 bead sets with peptides, as follows. Bead mix 1 contained 8 bead sets, loaded with resp. pCga001-003, pCga008-011, and NC; bead mix two contained 9 bead sets, loaded with resp. pCps001, 002, 009, 010, 013–016, and NC; bead mix three contained 10 bead sets, loaded with resp. pCab001-005, pCav001-004, and NC. The three bead mixes together constitute the Luminex suspension array when employed in parallel. By doing so, it was possible to run a cost-efficient multiplex assay for 24 peptides and internal controls with only 10 different beads.

### Performing the suspension array

Suspension arrays were performed using elements of existing protocols [18–20, 25] as follows. Sera were diluted 1:200 in sample buffer, i.e. PBS-T with 10% PRI-blocker, a dedicated blocking reagent for bead-based immunoassays (Prime Diagnostics, Wageningen, The Netherlands). To prevent background, diluted sera were also pre-treated with neutravidin (Thermo Fisher Scientific). In parallel, of the three different bead mixes, prepared in PBS-T with 10% PRI-blocker, 50 µL was mixed with 50 µL of pre-treated serum in a 96-well plate to a final serum dilution of 1:400, with 750 beads of each bead set present.

The three bead mixes were investigated in parallel as follows. The bead/serum mixes were incubated in a 96-well plate for 30 min in the dark at room temperature on a plate shaker, after which the plate was washed three times with 100 µL PBS-T, using a magnet (LifeSep 96F, Thermo Fisher Scientific) to precipitate beads. Next, 100 µL of phycoerythrin conjugated secondary antibodies in PBS-T was added (1:1000). For detection of bound serum antibodies, Goat Anti-Chicken IgY(H+L) (Southern Biotech) was used for chicken serum, whereas AffiniPure F(ab′)_2_ Fragment Goat Anti-Mouse IgG (H+L) (Jackson Immuno Research) was used for mouse serum. Beads were incubated with the secondary antibodies for 60 min in the dark at room temperature on a plate shaker. After washing, the fluorescence collected on the beads was measured with a Luminex LX200 system (Luminex), counting at least 75 beads per spectral region.

### Serum-specific cutoffs and normalization

To evaluate signals acquired on peptide beads, an approach was developed to establish serum-specific cutoffs, using the corresponding assay background observed for each individual serum on the negative control (NC) beads, that are present in each of the three bead mixes. To establish serum-specific cutoffs, first the specific assay background was calculated for each serum by averaging the median fluorescent signal (MFI) acquired on NC beads (avNC), present in the three bead mixes that were tested in parallel for each serum. Next, the specific cutoff was established for each serum by taking three times the corresponding assay background, i.e. three times the average MFI observed on NC beads (3*avNC), as established empirically (not shown) by studying the effect of different multiplicities of the internal negative control NC on agreement with the known status of the experimental sera. Resulting values were taken as serum-specific cutoffs and were used to normalize data by subtraction. For normalized data this resulted in a cutoff of 0 for each serum, which allowed comparison of signals obtained with multiple sera.

## Results

### Sera from experimentally infected mice confirm species-specificity of peptide beads

**Figure 1:**
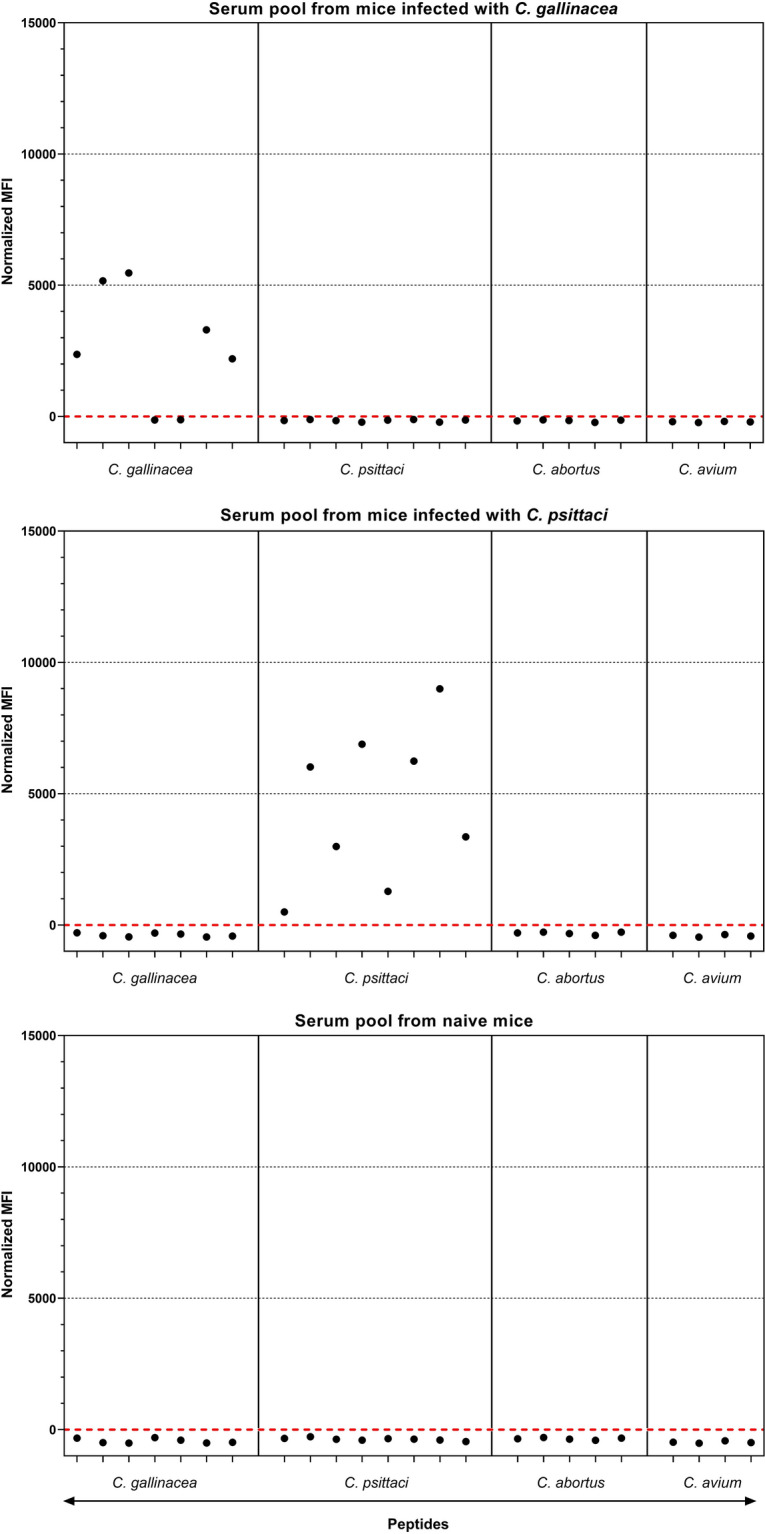
**Results of the suspension array with mice sera.** Results are shown for pooled sera from mice infected with *C. gallinacea* or *C. psittaci*, and naive mice. The three pools were tested with a fixed panel of beads carrying peptides or a negative control (NC) compound. Only signals on peptides are shown, expressed as normalized MFI (Y-axis) by subtraction of the serum-specific cutoffs that are based on signals on beads carrying NC. This resulted in a cutoff of 0 MFI for normalized data, represented by horizontal dashed red lines. Peptides are presented on the X-axis in the same order as listed in Table 1 and are labelled only as a group with the species names. See Additional file 1 for results (raw data) obtained with sera from mice infected with other *Chlamydia* species.

Results of the suspension array obtained with pooled sera from mice infected with *C. psittaci* or *C. gallinacea*, or from naive mice, are presented in Figure 1. With pooled serum from mice infected with *C. gallinacea*, only signals on beads with peptides derived from *C. gallinacea* exceeded the cutoff (Figure 1, top). Not all *C. gallinacea* peptides were seroreactive (see below). Signals on beads with *C. gallinacea* peptides remained below the cutoff when tested with serum from mice infected with *C. psittaci* (Figure 1, middle) or with serum from naive mice (Figure 1, bottom). Likewise, peptides derived from *C. psittaci* were specifically recognized by the serum pool from *C. psittaci* infected mice (Figure 1, middle). On all beads with peptides, signals remained below the cutoff when sera from naive mice were tested (Figure 1, bottom). Results for all available mouse serum pools (*n* = 18; Additional file 1, with raw MFI data) showed that most peptides derived from *C. gallinacea*, *C. psittaci*, *C. abortus*, and *C. avium* were recognized in a species-specific manner in the suspension array. Before normalization, signals above the serum-specific cutoffs ranged from 377 to 12 532 MFI, with most specific signals above 1000 MFI, whereas assay backgrounds (on NC beads) remained below 200 MFI, with standard deviations ranging from 0 to 23 MFI. These results agreed with those of the in-house ELISA in which low signals (OD < 0.1) were obtained with sera from naive mice whereas high signals (OD > 0.8) were obtained with sera from infected mice, including sera from mice infected with the four *Chlamydia* species focused on in this study.

In the suspension array, the signals on IncA peptides derived from the strain used for infecting mice, was always higher than on the orthologous IncA peptides derived from chicken isolates (Additional file 1). Of the IncA and OmpA *C. gallinacea* peptides derived from Dutch chicken isolates, the IncA-derived pCga002 orthologs (pCga010 and 011) were recognized by sera from *C. gallinacea* infected mice, in contrast to the two OmpA-derived pCga001 orthologs (pCga008 and 009) (see Additional file 1). Sequence differences between pCga002 (IncA) and its orthologs, and between pCga001 (OmpA) and its orthologs, can be seen in Table 1.

Cross-reactions with low signals were observed for one *C. avium* serum on two *C. psittaci* peptides (< 400 MFI; pCps002 and 014). Both are different length versions derived from the same region, and do not share homology with proteins from *C. avium* (BLAST). Cross-reactions with a low signal were also observed with two *C. pneumoniae* sera on an IncA-derived peptide (< 600 MFI; pCab001) that has only limited homology to proteins from *C. pneumoniae* (BLAST; lowest E-value 0.52 for 45% of the query).

Taken together, results with mice sera confirmed that *Chlamydia*-derived peptides can be used as antigens in a bead-based suspension array for detection of serum antibodies against multiple *Chlamydia* species and that biotinylated peptides retain their seroreactivity when bound to avidin-coated beads.

### Sera from chickens exposed to *C. psittaci* react with peptide beads in a species-specific manner

To investigate if the selected peptides react with sera from *C. psittaci* infected chickens, sera from three chickens exposed to *C. psittaci* were investigated. For these chickens, PCR data was available: early after the start of the experiment *C. psittaci* was detected in 2 out of 3 chickens (3031 and 3435); at the end of the experiment *C. psittaci* was detected in only one chicken (2829) (Additional file 2).

**Figure 2:**
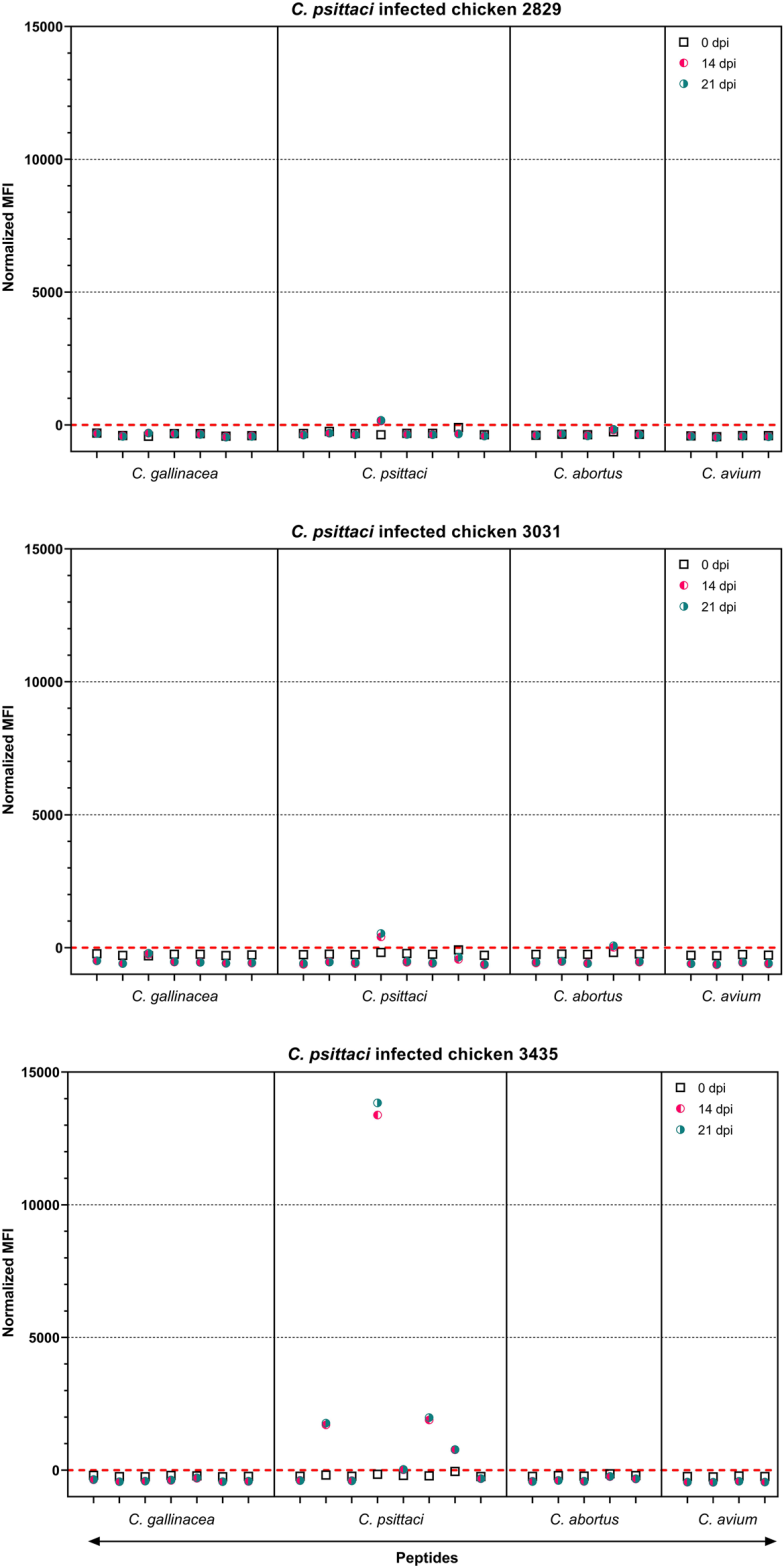
**Results of the suspension array with sera from chickens exposed to *C. psittaci*.** Results are shown for three chickens exposed to *C. psittaci* 6BC. Sera from day 0, 14, and 21 were tested (resp. black, red, and green symbols); for each chicken the results of three timepoints are presented in one graph. Signals on peptides are shown and are expressed as normalized MFI. For details see the legend to Figure 1. Raw MFI values are given in Additional file 2. dpi: days post-infection.

In the suspension array, the serum-specific assay background remained below 300 MFI for all sera, with standard deviations ranging from 3 to 45 MFI (Additional file 2, with raw MFI data). To visualize acquired signals on peptide beads of multiple sera in one graph, results were normalized by subtracting the calculated serum-specific cutoffs. By doing so, normalized signals were found negative on day 0 for all chickens, positive on day 14 and 21 for chicken 2829 and 3031 on one *C. psittaci* peptide (pCps010), or positive on day 14 and 21 for chicken 3435 on five *C. psittaci* peptides (incl. pCps010) (Figure 2). Three of the *C. psittaci* peptides were never recognized by sera from chickens exposed to *C. psittaci*. Of these, only pCps001 differed from the sequence of the strain used for infection (Table 2). All chicken sera from this experimental *C. psittaci* infection did not react with peptides derived from other *Chlamydia* species, apart from a cross-reaction for chicken 3031 with peptide pCab004.

**Table 2:** **Seroreactivity and sequence identity of** ***C. psittaci*-derived peptides**

*C. psittaci*-derived peptides	Blast results of peptide (query) against *C. psittaci* 6BC (subject)	Signal in infected animals	
		Mice	Chickens
		*C. psittaci* 02DC15	*C. psittaci* 6BC
pCps001_Cps_02DC15 OmpA_158-181	Query 1 LVGLIGFSAASSISTDLPTQLPNV 24	y	n
	Sbjct 158..................M..... 181		
pCps002_Cps_02DC15_IncA_329-352	Query 1 ADQGDLRDPSGDRYGGWGAQSSYR 24	y	y
	Sbjct 329........................ 352		
pCps009_Cps_02DC15_OmpA_250-265	Query 1 ASSNFPLPITAGTTEA 16	y	n
	Sbjct 250................ 265		
pCps010_Cps_02DC15_OmpA_329-352	Query 1 SLIGSTTALPNNSGKDVLSDVLQI 24	y	y
	Sbjct 329........................ 352		
pCps013_Cps_02DC15_PmpD_1053-92	Query 1 DPNAKPAEKIESPTSKVYYSAYDPVKNPGKKTLADINSIG 40	y	y
	Sbjct 1053........................................ 1092		
pCps014_Cps_02DC15_IncA_321-360	Query 1 SLTSTTETADQGDLRDPSGDRYGGWGAQSSYRLSPSVTMS 40	y	y
	Sbjct 321........................................ 360		
pCps015_Cps_02DC15_CT618_105-134	Query 1 YEVDSATGSFKIVTKNIQKPNGEVEIVSSR 30	y	y
	Sbjct 105.............................. 134		
pCps016_Cps_02DC15_CT618_189-228	Query 1 CGAVDDVISIVSTLRSTDFDPSYEDLVQRRVTLREKFFSL 40	y	n
	Sbjct 189........................................ 228		

For all *C. psittaci* 02DC15-derived peptides, the seroreactivity in the suspension array with sera from mice and chickens (infected with resp. 02DC15 and 6BC) is given. Alignments show the sequences of each 02DC15-derived peptide and corresponding sequences of 6BC. Identical amino acids are indicated with dots.

*y* yes, *n* no.

Results with the in-house ELISA showed increased signals on day 21 (day 14 sera were not tested) compared to day 0 for all exposed chickens, which was most pronounced for chicken 3435, i.e. the chicken that responded to five peptides (Additional file 3).

Summarized, all three chickens infected with *C. psittaci* had an increased response in the in-house ELISA at day 21 and recognized at least 1 *C. psittaci* peptide in the suspension array. In total five out of the eight *C. psittaci-*derived peptides (that are all recognized by mice sera) were recognized by chicken sera (Table 2). Signals on four out of these five peptides were obtained only with serum from chicken 3435, i.e. the animal with the lowest Ct value in a throat swab taken 4 days after exposure. These results showed that the suspension array is able to detect a specific response against *C. psittaci*.

### Sera from chickens infected with *C. gallinacea* react with peptide beads in a species-specific manner

**Figure 3:**
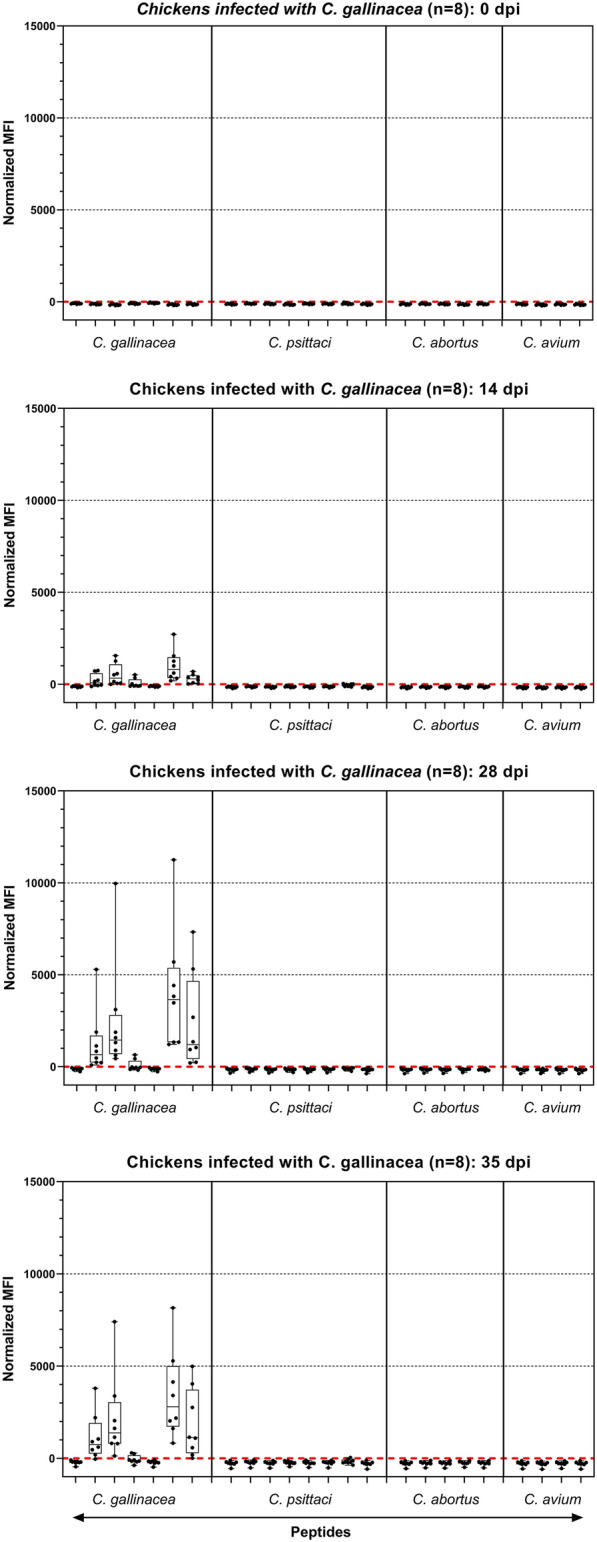
**Results of the suspension array with sera from chickens infected with *C. gallinacea*.** Results are shown for eight chickens infected with *C. gallinacea* NL_G47. Sera from day 0, 14, 28, and 35 were tested. For each peptide normalized signals are given for eight sera using a box-whisker plot. For details see the legend to Figure 1. Raw data are given in Additional file 4. dpi: days post-infection.

From eight chickens infected with *C. gallinacea* NL_G47, sera from day 0, 14, 28, and 35 were used for this study. In the suspension array, signals on all NC beads remained below 300 MFI. Normalized signals remained below 0 on all beads on day 0, and became positive after day 0 on a subset of beads with *C. gallinacea*-derived peptides (Figure 3).

All IncA-derived peptides with sequence homology to the published peptide pCga002 and its shorter variant pCga003 were recognized by the chicken sera, including the orthologs pCga010 and 011 derived from the two Dutch field strains (Table 3). For each chicken, the highest signal (see Additional file 4 with raw MFI data) on IncA peptides was always on pCga010, derived from the NL_G47 isolate that was also used for experimental infection of these chickens. The published OmpA-derived pCga001 peptide was never recognized, but its ortholog pCga008 from the Dutch field strain NL_G47 (used for infection) was recognized by a subset of the tested sera. The ortholog from another Dutch field strain, NL_F725, was never recognized. The differences in seroreactivity are listed in Table 3, as are the peptide sequences and the corresponding sequences of the infecting strains. Weak cross-reactions were observed with peptide pCps015 with some sera (see Additional file 4 for details).

**Table 3:** **Seroreactivity and sequence identity of** ***C. gallinacea*-derived peptides**

*C. gallinacea*-derived peptides	Blast results of peptide (query) against *C. gallinacea* NL_G47 (subject)	Signal in infected animals	
		Mice	Chickens
		C.gal. 08–1274/3 (08DC63)	C.gal. NL_G47
pCga001_Cga_08DC63_OmpA_326-345	Query 1 NPSFLGSADAQA––––TLVDSVQI 20	y	n
	Sbjct 328..T.S.G.VP.TGGTGSV..V... 351		
pCga002_Cga_08-1274/3_IncA_297-326	Query 1 SEAATSTSTPEGETSETKEGEEDSSVVEFD 30	y	freq
	Sbjct 297..........................I... 326		
pCga003_Cga_08DC63_IncA_303-326	Query 1 TSTPEGETSETKEGEEDSSVVEFD 24	y	y
	Sbjct 303....................I... 326		
pCga008_C.galG47_OmpA	Query 1 NPTFSGGAVPQTGGTGSVVDVVQI 24	n	incons
	Sbjct 328........................ 351		
pCga009_C.galF725_OmpA	Query 1 NPSFLGEANAQA----KLVDSVQI 20	n	n
	Sbjct 328..T.S.G.VP.TGGTGSV..V... 351		
pCga010_C.galG47_IncA	Query 1 SEAATSTSTPEGETSETKEGEEDSSVIEFD 30	y	y
	Sbjct 297.............................. 326		
pCga011_C.galF725_IncA	Query 1 SEAATSTSTPEGETSETKEGEEDSSVIDFD 30	y	y
	Sbjct 297...........................E.. 326		

For all *C. gallinacea*-derived peptides, the seroreactivity in the suspension array with sera from mice and chickens is given. Alignments show the sequences of the used peptides and corresponding sequences of strain NL_G47. Identical amino acids are indicated with dots.

*y* yes, *n* no, *freq* frequently (in all chickens, but signals are late, low, or transient), *inconsis* inconsistently (in 3 out of 8 chickens).

With the in-house ELISA it had been shown that sera from day 14, 28, 35 resulted in high signals, whereas sera from day 0 had low signals, indicating that sera taken post-infection contained *Chlamydia* antibodies [22]. In ELISA, signals were considerably higher for sera that were seropositive for *C. gallinacea* in the suspension array than for seronegative sera (Additional file 5).

Summarized, of the seven *C. gallinacea* peptides, two were recognized by all sera from chickens infected with *C. gallinacea* NL_G47, three were recognized but not consistently, and two OmpA-derived peptides from strain NL_F725 and 08DC63 were never recognized. Seropositivity as determined by the suspension array correlated with signal strength of signals obtained with the in-house ELISA. These results show that a subset of the *C. gallinacea*-derived peptides can be used to specifically detect antibodies against *C. gallinacea* in infected chickens, and also suggest that this subset can be used to differentiate between antibodies against *C. gallinacea* and *C. psittaci*.

### Field sera

A set of 120 field sera from layers and broilers with unknown infection status, were tested with the suspension array. The sera were from three types of chicken farms, with free range laying hens, conventional laying hens, or broilers, two farms each.

**Figure 4:**
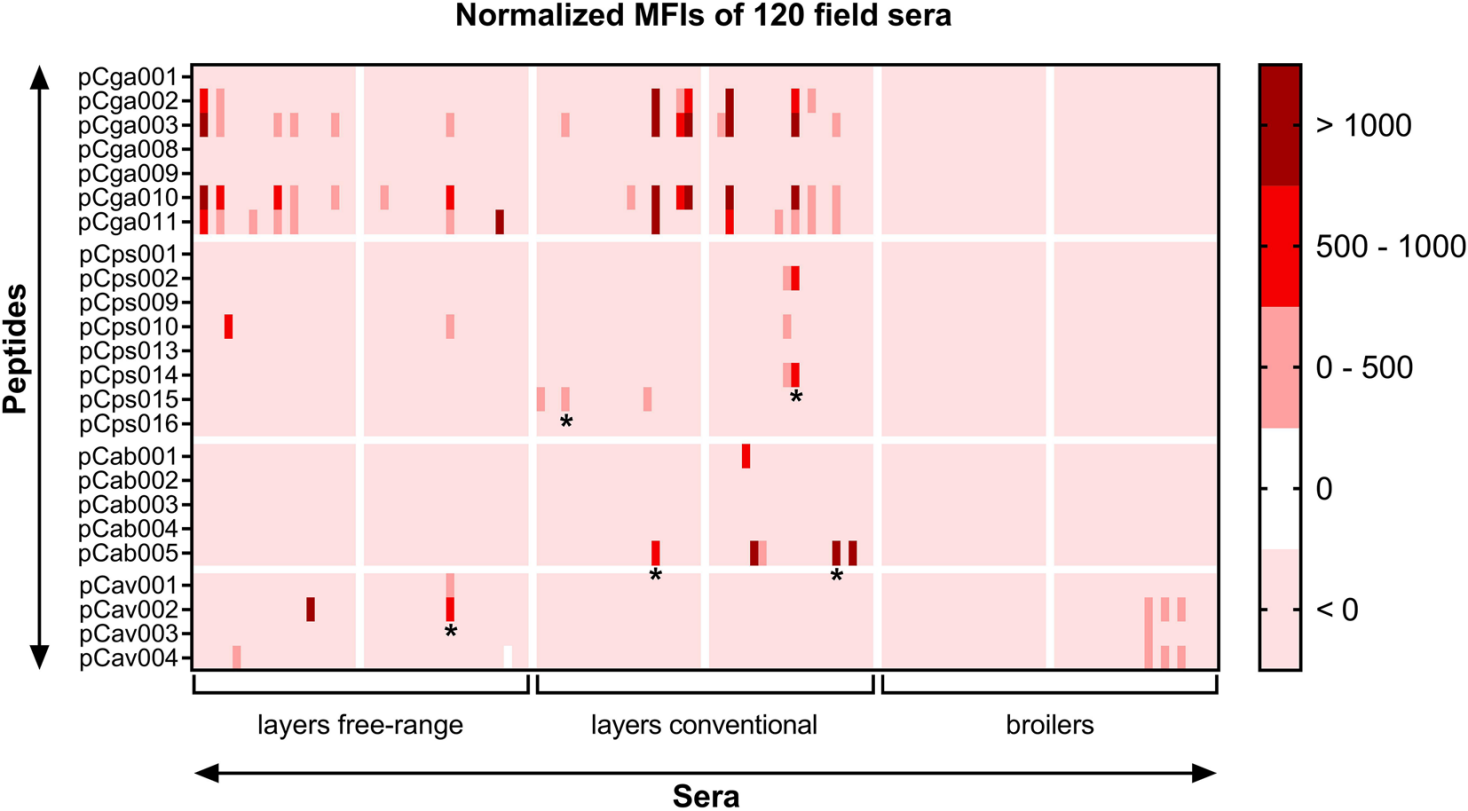
**Results for 120 field sera tested with the suspension array.** Normalized MFI values are given and categorized as indicated. Sera that are seroreactive toward peptides of more than one *Chlamydia* species are labelled with an asterisk below the last peptide recognized. Raw data are given in Additional file 6.

As for the experimental sera, for field sera the NC beads were tested simultaneously with the other peptide beads, resulting in information on assay background for each serum, that was used to normalize signals. In the suspension array, the background on the NC beads ranged from 115 to 2119 MFI in layers, and from 46 to 343 MFI in broilers. Sera were considered seropositive when a signal (positive after normalization) on at least one peptide was observed. This resulted in 33 seropositive sera, mostly from layers (Table 4). Of these, 20 sera were seropositive for *C. gallinacea*, and 19 for other *Chlamydia* species; 5 sera were positive for multiple *Chlamydia* species. The results are also presented in Figure 4 were seroreactivity of each serum is visualized (see Additional file 6 for raw MFI values).

**Table 4:** **Summarized results of field sera tested with the suspension array**

Peptides		Number of positive normalized MFI values				Positive sera per *Chlamydia* species^a^
Name	Description	Per peptide	Per farm type			
			Free range layers	Conventional layers	Broilers	
pCga001	Cga_08DC63_OmpA_326-345	0	22	28	0	20
pCga002	Cga_08-1274/3_IncA_297-326	8				
pCga003	Cga_08DC63_IncA_303-326	14				
pCga008	C. gal G47_OmpA	0				
pCga009	C. gal F725_OmpA	0				
pCga010	C. gal G47_IncA	15				
pCga011	C. gal F725_IncA	13				
pCps001	Cps_02DC15_OmpA_158-181	0	2	8	0	7
pCps002	Cps_02DC15_IncA_329-352	2				
pCps009	Cps_02DC15_OmpA_250-265	0				
pCps010	Cps_02DC15_OmpA_329-352	3				
pCps013	Cps_02DC15_PmpD_1053-92	0				
pCps014	Cps_02DC15_IncA_321-360	2				
pCps015	Cps_02DC15_CT618_105-134	3				
pCps016	Cps_02DC15_CT618_189-228	0				
pCab001	Cab_S26/3_OmpA_89-104	1	0	6	0	6
pCab002	Cab_S26/3_OmpA_153-176	0				
pCab003	Cab_S26/3_OmpA_158-173	0				
pCab004	Cab_S26/3_PmpD_1064-1087	0				
pCab005	Cab_S26/3_IncA_324-353	5				
pCav001	Cav_10DC88_IncA_305-328	1	4	0	7	6
pCav002	Cav_10DC88_IncA_299-328	5				
pCav003	Cav_10DC88_IncA_325-348	1				
pCav004	Cav_10DC88_IncA_319-348	4				
	Seropositive sera per farm type^a^:		12	18	3	

Results are presented for signals that in the suspension array exceeded the serum-specific cutoffs. Sera were from 120 chickens from three types of farm with resp. free range layers, conventional layers, and broilers.

^a^Of the 33 seropositive sera, 28 are seroreactive toward peptides of 1 Chlamydia species, 4 and 1 serum are seroreactive toward peptides of respectively 2 and 3 species.

In ELISA, signals for laying hens ranged from OD 0.19 to 1.36 for free range layers and from 0.10 to 1.01 for conventional layers, signals for broilers ranged from 0.00 to 0.132. The ELISA did not reflect the serostatus of individual sera as determined by the suspension array: the range of ODs of seropositives and seronegatives largely overlapped. However, for layers as a group the mean ODs were higher for seropositives than for seronegatives (Additional file 7).

In summary, field sera recognized subsets of *Chlamydia*-derived peptides, but the in-house ELISA and the suspension array did not agree with each other. In the suspension array, circa two thirds of the seropositive sera recognized *C. gallinacea*-derived peptides.

## Discussion

Current discriminatory tests for *Chlamydia* are based on detection of genetic material [13] and only give information about the actual infection status, but reveal nothing of past infections. In contrast, serological methods have a larger detection window. Detection of antibodies against *Chlamydia* has successfully been carried out with sera from various mammalian hosts [17]. The goal of the study presented here was to investigate if it is possible to develop a similar test for sera from chickens. In this study, focus was on *C. psittaci* and *C. gallinacea*, the two avian species that need to be distinguished, as one is zoonotic whereas the other currently is the prevalent species in chickens [10]. In addition, two genetically related species were included, i.e. *C. abortus* and *C. avium*, both occurring in birds [1], and the species that are most closely related to resp. *C. psittaci* and *C. gallinacea* [26]. To enable differentiating serology for the selected *Chlamydia* species, a bead-based Luminex suspension array was constructed, using peptides derived from immunoreactive *Chlamydia* proteins [15–17] as antigens. The suspension array correctly detected and identified antibodies against *Chlamydia* in sera from experimentally infected mice, showing that the peptides retained their seroreactivity in this assay format. Importantly, the test was able to differentiate between antibodies against *C. psittaci* and *C. gallinacea* in sera from experimentally infected chickens. Results with field sera confirm the high prevalence of *C. gallinacea* in layers. As yet, signals against peptides derived from other *Chlamydia* species are difficult to interpret as limited (*C. psittaci*) or no (*C. abortus*, *C. avium*) sera from experimentally infected chickens were available for evaluation.

Observations with infected chickens showed that upon *C. psittaci* infection specific antibodies were generated. Not all *C. psittaci* peptides were recognized, which may be an effect of the transient nature or inefficiency of the infection: PCR data showed that the *C. psittaci* infection of these chickens had been suboptimal and was transient. For *C. gallinacea* infected chickens there were no doubts concerning the infection, and signals in both ELISA and the suspension array were high upon infection.

It would be of added value to test additional sera from chickens infected with *C. psittaci* to investigate if the three non-reacting peptides can be recognized if chickens are not transiently infected.

An alternative explanation for the observed differences in seroreactivity of a subset of the *C. psittaci* peptides may be the dissimilarity of immune systems of mammals and birds, including differences in antibody development and antigen processing [27–29]; mice and birds may recognize different epitopes. This speculation is fed by two observations. Of the *C. psittaci*-derived peptides that are only seroreactive in mice, two have the same sequence as the 6BC isolate used for infecting chickens, yet do not result in a signal in the suspension array. Further, sera of *C. gallinacea* G47 infected chickens recognize (but inconsistently) the autologous G47 OmpA-derived peptide pCga008, whereas pooled sera from mice infected with *C. gallinacea* do recognize the autologous peptide, pCga003 that is based on the same region, resulting in a high signal.

With sera from infected mice some cross-reactions were observed, that could not be traced back to sequence homology and were not observed before in the peptide array [17], but one of these cross-reactions has been observed before in ELISA (pCab1, Cab_S26/3_OmpA_89-104) [16]. Since the signals of cross-reactions are low in the suspension array and low or absent in other platforms, it is likely that differences in designated serostatus of sera with low signals, are caused by differences in the selected cutoffs that are used. Also with sera from experimentally infected chickens some cross-reactions were observed. One *C. psittaci* infected chicken reacted with peptide pCab004, that has 71% identity with its ortholog in *C. psittaci* 6BC (BLAST). It’s sequence overlaps with a published peptide which is known to cross-react in ELISA with sera from mice infected with *C. psittaci* [16]. A subset of sera from the *C. gallinacea* infected chickens also cross-react with *C. psittaci* peptide pCps015, for which no explanation was found since the peptide has only limited homology to proteins from *C. gallinacea* (BLAST; lowest E-value 0.97 for 36% of the query). Regardless of the nature of these cross-reactions, in order to provide straightforward interpretation of results, it shows that panels of strong reacting peptide antigens for each species have a preference.

In this study, a generic method was developed for establishing a cutoff for each serum sample. In antibody detection, it is common practice to base cutoffs on the assay background or the average signal of a negative control group of animals, plus two or three times the standard deviation [25, 30]. Here, an approach was adopted to base a cutoff on a negative population of beads within each assay by defining a serum-specific cutoff for each individual serum sample, that is based on the individual assay background of that serum, which in singleplex platforms such as ELISA is not possible for individual samples. This serum-specific assay background was measured using internal negative control (NC) beads that were present in each bead mix. These control beads carry a compound similar to the peptides used, but without an actual peptide moiety and are expected to reflect aspecific binding of serum components in the system. Of these, in each well at least 100 beads were analysed—these are regarded as the negative population of beads within each assay and are thus used as the basis of calculating a cutoff for each serum. When analysing all results, the serum-specific assay background times three was found acceptable as cutoff for all experimental sera. This method of calculating individual serum-specific cutoffs takes away the assay background, resulting in limited aspecific reactions and/or cross-reactions. The cutoff can not take into account cross-reactions of particular peptides; by the time a fully developed assay is available, a comparison with a negative population of animals will help in both finetuning the cutoff and assembling a final peptide panel.

The approach with a universal method to establish serum-specific cutoffs was also employed for analysing field sera, but was not verified with a large set of chicken sera that are seronegative according to a reference test, as these are simply not available. Investigation of 120 Dutch field sera with the suspension array confirmed that *C. gallinacea* is the prevalent *Chlamydia* species in layers, which agrees with observations made using PCR [10], and also showed that *C. gallinacea* antibodies are absent in broilers, at least in the 40 sera tested. The results suggest that the current test may be used to establish if flocks are seropositive for *C. gallinacea*. Signals against peptides derived from other *Chlamydia* species than *C. gallinacea* were observed in mainly laying hens, but are difficult to interpret as limited (*C. psittaci*) or no (*C. abortus*, *C. avium*) sera from experimentally infected chickens are available to evaluate the seroreactivity of these peptide panels. So, the seroreactivity of peptides from species other than *C. gallinacea* was clearly demonstrated with sera from experimentally infected mice, but sera to do so unambiguously for chickens are not available. Although some information was acquired with sera from immunizations of chickens with *C. psittaci* and *C. abortus* bacterins, such information is not available for *C. avium* (see Additional file 8).

The observation that of these peptide panels the field sera recognized only subsets or individual peptides may indicate presence of specific antibodies, but these “sporadic” signals may also be aspecific interactions or cross-reactions with antibodies against unidentified pathogens. Some peptides cross-reacted in experimental chicken sera (e.g. pCps015) and with field sera probably have no relevance. A peptide such as pCab005 was recognized by field sera as solitary signal or coinciding with signals on *C. gallinacea* peptides. However, this peptide did not cross-react with experimental *C. gallinacea* sera, nor was it the strongest responder in the *C. abortus* peptide panel when tested with mice serum. A complicating issue here is that there is no data on the presence of *Chlamydia* species/strains (sequence variants) in the sampled chickens to verify the serological signals observed. Another complication is the aforementioned lack of experimental chicken sera for all *Chlamydia* species (also see Additional file 8).

Concerning possible sequence variants, for *C. gallinacea* IncA-derived peptides signals were always highest on the autologous versions of these peptides when testing with experimental sera. In field sera however, for each of these three peptides sera exist that are only positive on one variant. This may suggest that multiple *C. gallinacea* strains occur in the set used.

Regarding comparisons of the results of the ELISA and the suspension array, it is remarkable that with field sera there is no clear correlation between the two assays, whereas for sera from experimentally infected mice and chickens the results of the two assay do agree, i.e. with sera from experimental infections in both assays low signals are observed prior to infection, and high signals are observed after infection. Nevertheless, many field sera show high signals in ELISA and are not seropositive in the suspension array. An explanation may be related to the nature of the antigens. The in-house ELISA is performed with a mixture of inactivated *C. abortus* and *C. trachomatis* bacteria, in fact elementary and reticular bodies containing a multitude of antigens, which may in part be responsible for causing cross-reactions. One type of cross-reaction is within the group of C*hlamydia* species. In ELISA this antigen mixture enables detection in animal sera of antibodies against all other *Chlamydia* species, as demonstrated in this study with sera from experimentally infected mice and chickens. This is consistent with published observations where such antigens are shown to be seroreactive with antibodies against (all) other *Chlamydia* species [31]. The broad nature of the ELISA antigens may also be responsible for another type of cross-reactions: cross-reactions by antibodies against other bacteria that occur in chickens, *Chlamydia*-related or otherwise. Cross-reactions with other *Chlamydia* species than studied here is unlikely since *C. gallinacea* is the prevalent species in Dutch poultry [10], and there is no direct evidence for cross-reactivity of antigens between *Chlamydia*-related species (in mammalian sera) if they are not closely related [32]. The observation that in production animals ELISA signals are always low in young animals (broilers, aged between 4 and 6 weeks) but can be very high in older chickens (layers, in general 18 weeks or older) is indicative of an age-related presence of antibodies; older animals on farms undoubtedly have encountered more bacteria than young chickens or chickens used for experimental infections. Another, simple, explanation could be that the peptide panel is simply missing essential peptides, but if it is, that is only a part of the problem as the observed large number of layers seropositive for *C. gallinacea* match the prevalence found by PCR [10], a study in which no other *Chlamydia* species were detected. A complicating factor in this whole discussion is that for the ELISA cutoff has not been established [21]. For now, the mismatch between results from the in-house ELISA and suspension array remains elusive.

The value of having serum-specific cutoffs was shown when testing field sera. For two field sera, extremely high assay backgrounds were observed in the suspension array (> 1000 MFI on NC beads). For one serum, this was also reflected by high signals on other beads, whereas the standard deviation (SD) for the NC beads (measured in three parallel assays) was low (38 MFI). Despite the high serum-specific assay background, and hence the high cutoff, this serum had only three signals that exceeded the cutoff, and was designated seropositive for *C. gallinacea* with seroreactivity on the same peptides as seen for many other sera. For the other serum, the high serum-specific assay background was reflected by many, but not all beads, and the SD NC was also high (237 MFI). Although this serum was designated seronegative, it clearly is an aberrant serum with unusual behaviour toward the control beads. In the experimental setup with three parallel tests this was visible (NC beads are used in each parallel test), but in a complete multiplex test however this would go unnoticed as all beads would be tested in one well. So, an additional rule to filter out sera with an aberrant/irregular background signal could improve the suspension array. Summarized, these observations show the value of a serum-specific cutoff as it corrects for high assay background, but also suggest that some improvements for sera with an irregular background may be required.

The developed serological Luminex suspension array described in this work shows great promise as it is able to discern in experimentally infected chicken sera if antibodies against *C. psittaci* or *C. gallinacea* are present, and confirms that *C. gallinacea* is the prevalent *Chlamydia* species in layers. The protocol to produce beads with synthetic peptides is simple, and performing a multiplex assay with a panel of peptides is uncomplicated and not troubled by background issues, the latter in part facilitated by using negative control beads that allow individual cutoffs for each serum.

To develop this proof of concept assay into a full multiplex assay for detection and differentiation of antibodies against *Chlamydia* species, a panel of peptides is required that covers multiple antigenic regions of the targeted species, either conserved linear epitopes or a panel covering sequence variants. These can be obtained from the relevant publications [15–17], a new inventory of the ever increasing number of genome sequences, and/or by (re)screening antigenic proteins using arrays with overlapping peptides. To do this, additional chicken sera are required that are experimentally infected with the various *Chlamydia* species of interest. Further, for validation a set of paired samples should be available from a longitudinal study in poultry that allow detection and identification of the infecting *Chlamydia* species by PCR and evaluation of peptides by serology.

## Supplementary Information

**Additional file 1.** **Results of the suspension array with sera from mice infected with various *Chlamydia* species.** Pooled sera from mice experimentally infected with *Chlamydia*, and two pools from naive mice, were tested with bead sets loaded with antigens as indicated. Results for *C. psittaci*, *C. gallinacea*, and the last naive mouse are also presented in Figure 1. The results are given in MFI, values above the cutoffs are highlighted in red. For beads with the negative control (NC) compound, values given are the average MFI of three parallel runs; the standard deviations are also given (SD). Serum-specific cutoffs were calculated as three times the average signal on NC beads from three parallel runs (3*avNC). Abbreviations: p, purified peptide; Cga, Cps, Cab, and Cav resp. *C. gallinacea*, *C. psittaci*, *C. abortus*, *C. avium*. NB in the manuscript normalized data are used for which the cutoffs were subtracted from the acquired MFI values.

**Additional file 2.** **Results of the suspension array with sera from chickens infected with *C. psittaci* and the available PCR data.** Sera from three chickens experimentally infected with *C. psittaci* 6BC were tested with a fixed panel of beads carrying the antigens indicated in the first column. Results are given in MFI. Values above the cutoffs (3 times the signal on NC beads) are highlighted in red. The same results are presented in Figure 2, but there MFIs were normalized by subtracting 3xavNC to enable visualisation of data for multiple sera in one graph. Abbreviations: dpi, days post-infection; for other abbreviations see Additional file 1. NB in the manuscript normalized data are used for which the cutoffs were subtracted from the acquired MFI values. Ct values for the three chickens exposed to *C. psittaci* 6BC are given for 4 and 21 dpi for swabs and tissue samples as indicated. Ct values that are regarded positive are marked red.

**Additional file 3.** **Comparison of ELISA and suspension array for *C. psittaci* infected chickens.** To allow comparison of the suspension array with the in-house ELISA, for which no cut-off is available, sera from chickens experimentally infected with *C. psittaci* were designated seropositive for *C. psittaci* when a positive signal on at least one *C. psittaci* derived peptide was observed. By plotting signals (OD) from the in-house ELISA against the two categories (seropositive and seronegative in the suspension array), the relation between the assays was visualized. Symbols: triangles, chicken 2829; circles, chicken 3031; squares, chicken 3435; open symbols, seropositive in suspension array; closed symbols, seronegative in the suspension array; grey line, mean OD.

**Additional file 4. Results of the suspension array with sera from chickens infected with *C. gallinacea*.** Sera from 8 chickens experimentally infected with *C. gallinacea* NL_G47 were tested with a fixed panel of beads loaded with antigens as indicated in the first column. These data were also used for Figure 3, but there MFIs were normalized by subtracting 3xavNC. Here, results are given in MFI, values above the cutoffs are highlighted in red. For abbreviations see Additional files 1 and 2. NB in the manuscript normalized data are used for which the cutoffs were subtracted from the acquired MFI values.

**Additional file 5. Comparison of ELISA and suspension array for *C. gallinacea* infected chickens.** To allow comparison of the suspension array with the in-house ELISA, for which no cut-off is available, sera from chickens experimentally infected with *C. gallinacea* were designated seropositive for *C. gallinacea* when a positive signal on at least one *C. gallinacea* derived peptide was observed. By plotting signals (OD) from the in-house ELISA against the two categories (seropositive and seronegative in the suspension array), the relation between the assays was visualized. Symbols: closed circles, seronegative in the suspension array; open circles, seropositive in the suspension array; grey line, mean OD.

**Additional file 6.** **Results of the suspension array with 120 chicken field sera.** Sera from 120 chickens, 20 each from two farms of three farm types, were tested with a fixed panel of beads, loaded with antigens as indicated in the first column. These data were also used for Table 4 and Figure 4. The results are given in MFI, values above the cutoffs are highlighted in red. For a description of cutoffs and abbreviations see Additional file 1. NB in the manuscript normalized data are used for which the cutoffs were subtracted from the acquired MFI values.

**Additional file 7.** **Comparison of ELISA and suspension array for field sera.** Using the serum-specific cutoffs defined for the suspension array, field sera were designated seropositive or seronegative. ELISA results (OD) were plotted against the various categories (farm type + serostatus in the suspension array). Symbols: closed circles, seronegative in the suspension array; open circles, seropositive in the suspension array; grey line, mean OD.

**Additional file 8.** **Seroreactivity of *C. psittaci* and *C. abortus* derived peptides with sera from immunized chickens.** In addition to observations on seroreactivity of peptides derived from *C. gallinacea* and *C. psittaci*, immunizations were performed with bacterins of home-grown *C. psittaci* and various commercial *Chlamydia* preparations (Virion/Serion); material of *C. avium* was not available. At the start of the experiment all chickens showed no signals exceeding the cutoffs, except chicken 145 (*n* = 1) and 172 (*n* = 2), where the signals by serum from chicken 172 were relatively high (> 1000). In the final bleeds of chickens immunized with *C. psittaci* bacterins, antibodies were present against a subset of *C. psittaci* peptides. Two out of four of these chickens also showed reactivity to one *C. abortus* peptide, one chicken showed a very low cross-reactivity toward a *C. gallinacea* peptide. The immunization with *C. abortus* bacterin in one chicken resulted in high signals on two *C. abortus* peptides. This suggests that chickens are capable of an immune response to at least a subset of the *C. abortus* peptides. Both *C. abortus*-immunized chickens showed also high signals on two *C. avium* peptides in the final bleeds: this cross-reactivity was not observed in infected mice (Figure 1, Additional file 1) and also not in field samples (no coincidence of signals against *C. abortus* and *C. avium* peptides, Figure 4). Chickens immunized with bacterins of two other species (*C. pneumoniae* and *C. trachomatis*) only in one case gave a modest signal with one *C. psittaci* peptide. In addition, chicken sera from the same experiment as the *C. psittaci* sera used for Figure 2, that were immunized after an ineffective infection, resulted in a specific immune response to most *C. psittaci* peptides. The panel recognized is broader than what is seen upon an infection experiment (Figure 2, Additional file 2). Taken together these results provide some additional evidence for seroreactivity of a subset of peptides in chicken sera. Observed cross-reactivities may suggest that the immune response upon immunization is different from that upon an infection: signals are high, more peptides are recognized, and even some very significant cross-reactivity is observed (with *C. avium* peptides).

## Data Availability

Data used in the manuscript for tables and figures are presented in accompanying additional files.
